# Can tendon reflexes be elicited by both stretch and vibration in man?

**DOI:** 10.1002/brb3.2191

**Published:** 2021-05-27

**Authors:** Peer Tfelt‐Hansen, Pirgit Meritam Larsen, Ulla van Deurs, Martin Fabricius

**Affiliations:** ^1^ Department of Neurology Rigshospitalet University of Copenhagen Glostrup Denmark; ^2^ Department of Clinical Neurophysiology Rigshospitalet University of Copenhagen Glostrup Denmark

**Keywords:** deep tendon reflexes, hyperreflexia, phasic vibration, stretch, supination

## Abstract

**Aim of study:**

When the biceps tendon is tapped, a contraction is elicited in the biceps muscle. This also occurs with tapping of the radial bone, and it has been suggested that vibration is a stimulus for deep tendon reflexes. We investigated whether the normal stimulus for the deep tendon reflex is a sudden stretch, a phasic vibration, or both. Furthermore, we investigated the importance of forearm position for the reflex response in controls and stroke patients.

**Methods:**

We investigated 50 neurological outpatients without clinical signs of neurological disorders in the arms. The biceps tendon and distal radius were tapped with the forearm in the midway (90°), supinated, and pronated positions. In 10 of these patients, the two reflexes were also investigated with quantitative electromyography (EMG) measurements in the 3 positions. Another 10 patients were investigated clinically when stretch of elbow was eliminated and 17 patients were examined when prestretching of the biceps tendon was avoided. Finally, we examined 32 patients that had experienced stroke.

**Results:**

In 94% (47/50) of patients, after a radial tap, the biceps contraction disappeared in the supinated forearm, and the median peak‐to‐peak amplitude of the surface EMG response (*n* = 10) decreased from 1.1 to 0.2 mV (*p* < .01). Elimination of elbow stretch as well as pressure on the biceps tendon did not change the reflex response. In 84% (27/32) of stroke patients, after a radial tap, the biceps contraction persisted in supination in the arm with hyperreflexia.

**Conclusion:**

The combined clinical and EMG results are consistent with the concept that the deep tendon reflexes in man can be elicited by both stretch and phasic vibration. Clinicians should be aware that the brachioradial reflex depends on the forearm position.

## Graphical abstract

Human reflexes; Position of upper limb; Stroke.

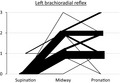

## 1 INTRODUCTION

“A tap on a tendon stretches or perhaps causes vibration of the spindle and activates its nuclear bag fibers.” Citation from *Adams and Victor's* *Principles of Neurology*, 10th Edition, 2014 (Ropper et al., 2014)

The biceps reflex is called a deep tendon or a muscle stretch reflex. The reflex contraction in the biceps muscle can be elicited by several, quite different examination techniques, including.

1. Tapping the tendon of the biceps muscle (Campbell, 2005).
2. Eliciting the brachioradial reflex; that is, tapping the distal part of the radius at the brachioradial muscle insertion; this results in contraction of the brachioradial muscle, but usually, the biceps muscle also contracts (Wartenberg, 1945).
3. Eliciting other “irradiating reflexes”; for example, tapping the vertebral border of the scapula (the scapulo‐humeral reflex) can result in a contraction of the muscles around the shoulder, and occasionally, the biceps muscle (Wartenberg, 1945).

Additionally, many animal and human studies have shown that a sudden stretch could elicit deep tendon reflexes (Boes, 2014; Hayashi et al., 1987; Karam, 1977; Liddell & Sherrington, 1924; Marsden et al., 1976; Shemmell et al., 2010; Stam & Crevel, 1969). Wartenberg maintained that “irradiating reflexes” were also due to stretching the muscles (Wartenberg, 1945). The primary spindles of muscles are extremely sensitive to tonic (i.e., sustained) vibration (Bianconi & Van den Meuelen, 1963; Echlin & Fessard, 1938; Grant & Henatsch, 1956), and they are also possibly sensitive to phasic (i.e., brief) vibration. When ischemia blocks conduction in the large fast‐conducting fibers of nerves in the forearm, the brachioradial reflex remains intact (Lance, 1980); thus, a tap on the radius will still elicit a biceps muscle contraction (Lance & de Gail, 1966). Based on this and other observations of the “irradiating reflexes,” in 1980, Lance concluded that “The propagation of vibration along a taut tendon and muscle fiber is probably the mechanism of producing a normal tendon jerk” (Lance, 1980).

Accordingly, two different opinions prevail concerning the stimulus for eliciting deep tendon reflexes in man. Here, we re‐evaluated the clinical features of the brachioradial and biceps reflexes, combined with a neurophysiological study, to elucidate whether the normal stimulus for the deep tendon reflex was a sudden stretch, a phasic vibration, or both.

A peculiar feature of the brachioradial reflex is that it disappears when the forearm is in full supination (Dumpert & Flick, 1922; Wartenberg, 1945). In contrast, this does not occur with the biceps reflex (Wartenberg, 1945). We compared this reflex in normal subjects and patients that had experienced a stroke to determine whether this phenomenon could be a useful clinical sign.

### 1.1 Hypotheses

1. Elimination of elbow stretch will not affect the brachioradial reflex if vibration is an adequate stimulus.
2. Eliciting the biceps reflex, without keeping it taut by depressing the tendon first, will not affect the biceps reflex if stretch is an adequate stimulus.
3. Supination of the forearm will eliminate the brachioradial reflex in controls, but not in a condition of hyperreflexia.

## 2 METHODS

### 2.1 Patient recruitment and groups

Altogether we studied 109 different individuals, 32 of these were stroke patients (see below), and 77 were outpatients from the Department of Neurology, Rigshospitalet, Glostrup, Denmark. These 77 patients had no signs of first motor neuron disease or a peripheral neurological disorder. They were selected for the study based on a screening procedure that was part of the routine clinical examination performed by one investigator (PTH). Briefly, the brachioradial reflex was elicited with the patient in the sitting position. The radius was tapped, while the patient's hand was resting in the lap and the forearm was turned in a midway position, between pronation and supination. All included patients responded to the tapping with a visible contraction of the biceps muscle on at least one side. Of these 77 patients, 50 patients constituted the main study group, ten of these patients were investigated with EMG in addition to the clinical examination of reflexes, and 20 of these patients were examined clinically twice to test for intraobserver variations. To study specific details, we subsequently included ten other patients to study effect of eliminating movement in the elbow joint and finally another 17 patients to study the effect of compressing the biceps tendon versus a slight touch only before striking with the hammer.

We also included 32 patients that were either inpatients or outpatients at the Stroke Unit, Department of Neurology, Herlev‐Gentofte Hospital, Denmark. Patients had experienced an acute stroke (*n* = 16) or a previous stroke more than 3 months ago (*n* = 16). These patients were selected based on the observation that the brachioradial reflex was more brisk on the affected arm than on the healthy arm.

### 2.2 Ethical considerations

This study was approved by the Science Ethics Committee in the Capital Region of Denmark (Protocol no. H‐1–2012–038) and by the Danish Data Protection Agency (Protocol no. 2007–58–0015). All patients that were eligible upon routine clinical screening were given written and verbal information by one investigator (PTH). All included patients provided informed, written consent.

### 2.3 Technique for eliciting reflexes

Reflexes were elicited by a brisk tap with a standard rubber reflex hammer. The brachioradial reflex was elicited by tapping the radius, 4–5 cm proximal to the tip of the styloid process. Fingers II and III of the investigator were interpositioned as a pad. The biceps reflex was elicited by tapping the tendon of the biceps muscle, with the investigator's finger II placed as a pad on the tendon and exerting a light pressure (notably, 17 patients were also tested without pressure). Eliciting the reflexes was done by one investigator (PTH) with a brisk tap on the fingers that could still be tolerated by the investigator.

### 2.4 Rating the clinical reflex response

Reflexes were tested and rated by one investigator (PTH). Ratings were based on observing the biceps muscle and elbow flexion, if present, as follows: 0 (0) = no visible contraction, no elbow flexion; + (1) = visible contraction, with no or discrete elbow flexion; ++ (2) = clearly visible contraction and visible, but moderate elbow flexion; +++ (3) = clearly visible contraction and extensive elbow flexion. This is a modification of the National Institute of Neurological Disorders and Stroke (NINDS) Muscle Stretch Reflex Scale (Hallett, 1993).

### 2.5 Quantitative measurement of EMG response

EMG measurements were performed on 10 patients of the main control group. Patients were examined in a sitting position on a bench. The forearm (angle of the elbow was 90°) was placed on a custom‐made table. Wooden bars mounted on the table eliminated movements in any downward or sideward direction. Measurements were performed consecutively, with the forearm in the midway, fully pronated, and fully supinated positions.

EMG responses were recorded with adhesive surface electrodes (Neuroline). The active electrodes were placed on the most prominent muscle bellies of the brachioradial, biceps, and triceps muscles. The reference electrodes were placed over the insertion tendons of the brachioradial and triceps muscles, and on the acromion, for the biceps muscle. The vibration of the distal humerus in response to the tapping was measured by a 15 mm long piezoelectric electrode (Dantec) taped to the skin over the lateral humerus epicondylus; the voltage induced by the vibration of the electrode was recorded together with the EMG response.

The radial bone and the biceps tendon were tapped with a special tendon reflex hammer (Dantec), attached to a cable for triggering the EMG sweep. The EMG responses were measured with a four‐channel Keypoint electromyograph (Natus) and Keypoint.net software. The filter was set to a 20 − 10 kHz bandwidth; the sampling rate was 48 kHz, and a full sweep lasted 100 ms. Repeated tapping was performed at each position, with at least 30 s between taps, to obtain at least three reproducible responses, and measurements were done on the first of these responses. Responses marred by movement artifacts or insufficient tapping were discarded.

Latencies were assessed by measuring traces on a graph with 0.5 mV/div for amplitudes and a 5 ms/div for time. Latency was assessed from the tapping to the first reproducible deflection. We also assessed the peak‐to‐peak amplitude responses of the polyphasic compound surface EMG. The vibration signal obtained by the piezoelectric electrode started within a few ms after the tapping, and maximal peak‐to‐peak amplitude was measured within the first 10 ms of the trace.

### 2.6 Statistics

Continuous data are expressed as the median (range); these data are compared between groups with Wilcoxon's test for paired data (socscistatististics.com). Discrete data are expressed as the mean (range); these data are compared between groups with Fisher's exact test (vassarstats.net). Here, we use the mean, because often, in a 4‐step scale, the median would not reveal differences.

For each arm and reflex, peak‐to‐peak amplitude of the vibration‐response was compared between the three positions of the forearm as % of the mean of these three responses.

A statistical significance threshold of 0.01 was chosen, due to multiple comparisons.

## 3 RESULTS

### 3.1 Clinical investigations

#### 3.1.1 Routine reflex investigation

We studied 50 patients (34 females and 16 males; median age: 51 years, range: 26–84 years) without neurological disorders involving the upper extremities. The three most common diagnoses were migraine, with and without aura (26%), no neurological diagnosis (16%), and cluster headache (11%). These patients were tested with the hand in the lap. Ten of these patients were also investigated with EMG.

**FIGURE 1:**
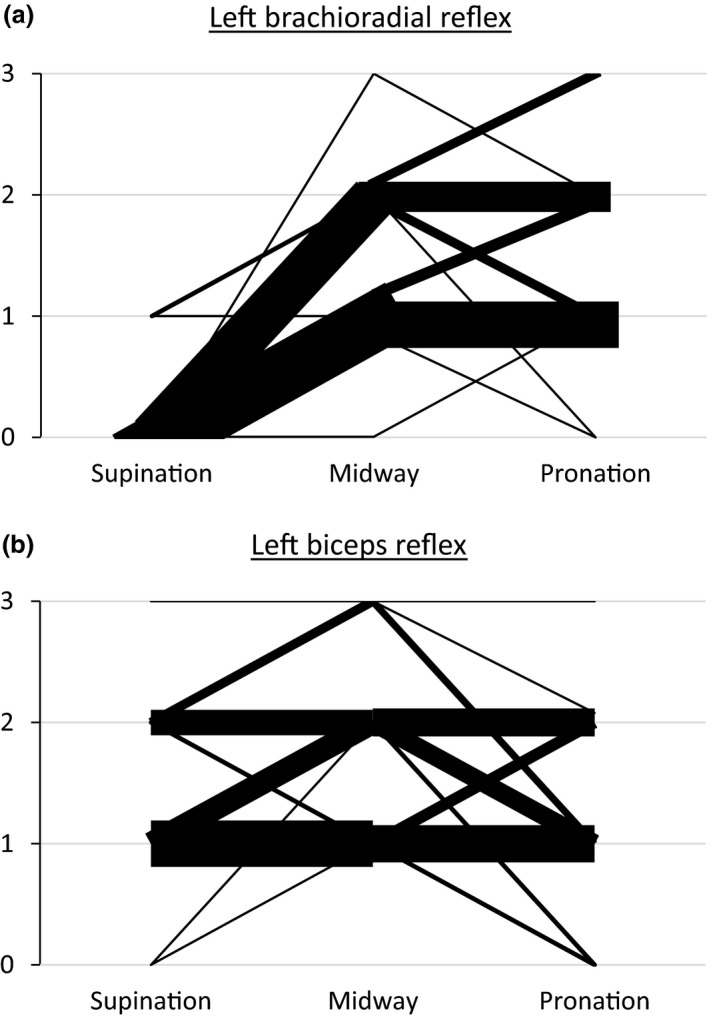
(a) Effect on the brachial reflex of position of forearm, midway (90^0^), supination, and pronation. (b) Effect on the brachial reflex of position of forearm, midway (90^0^), supination, and pronation. 1: Change in clinical response depending on forearm position. 50 persons with no signs of first motor neuron disease or a peripheral neurological disorder were tested. A: Brachioradial reflex and B: Biceps reflex. Position of the forearm was either in full supination, midway, or full pronation. Response was assessed clinically as: 0: no visible contraction in the biceps. 1(+), visible, but no flexion. 2(++): moderate elbow flexion. 3(+++): extensive elbow flexion. The changes are depicted by bars, the width of these represent the number of individuals presenting that particular change, for example, 25 persons in 1A was scored as 1(+) in midway position and 0 in supination, but had various responses in full pronation, while only one person showed 0 response in both supination and midway, yet 1(+) in full pronation

The results for the left arm (right arm results were quite similar, not shown) are shown in Figure 1a and b depicted as bars showing the individual changes of biceps contractions (0, +, ++, and +++, see Methods) after supination and pronation for the brachioradial and biceps reflexes. With the left forearm in the midway (90°) position, we first tested the brachioradial reflex. A radial tap elicited a visible contraction of the biceps muscle in 49 patients, and no response in one patient. Among the 49 patients, when the forearm was placed in full supination, the contraction in response to a radial tap disappeared in 46 patients and was still present in 3 patients (Figure 1) *p* <.001). When the forearm was pronated, the contraction in response to a radial tap disappeared in 2 patients and was still present in 48 patients (Figure 1). Mean change was −1.4 for midway to supination and 0.04 for midway to pronation.

The contractions of the biceps muscle to a radial tap thus disappeared in 94% of the left forearms in supination, whereas this only occurred in 4% after pronation (*p* <.001).

Next, we tested the biceps reflex. With the forearm in the midway position, a tap on the biceps tendon elicited a visible contraction. When the left forearm was placed in supination the contraction response of the biceps muscle disappeared in 2 of 50 (4%) of patients, whereas in pronation the response disappeared in 4 out of 50 (8%) patients (NS; Figure 1b).

To test for intraobserver variations, 20 patients of the main study group were investigated twice for brachioradial and biceps reflexes in the 3 positions. The left arm reflexes were scored twice with 1–2 min in between. The reflexes were scored as mentioned above by one investigator (PTH). In supination, the brachioradial reflex was always 0 in both examinations. Generally, the brachioradial and biceps reflexes gave identical values between the two trials in 16 of 20 patients, but in four patients a few scores differed by one point (NS). We conclude that there was a high intraobserver reproducibility.

#### 3.1.2 Hand in lap versus forearm and hand stabilized on a custom‐made table

In 10 other patients (7 females, 3 males; median age 50, range: 26–60 years), we examined the reflexes with either the hand in the lap or with the forearm on a table. For the left arm in the midway position, the mean (range) brachioradial response scores were 1.2 (0–2) with the hand in the lap and 0.9 (0–1) with the forearm on the table. In full supination, the response was 0, in all cases and in both positions. In full pronation, the mean response scores were similar in the lap (1.2 (0–2) and on the table (1.1 (0–2). For the biceps reflex, the responses in the midway position were 1.4 (1–2) in the lap versus. 1.2 (1–2) on the table; in full supination, the responses were 1.0 (0–2) versus. 0.7 (0–1), respectively; and in full pronation, the responses were 1.2 (0–2) versus. 1.0 (0–2), respectively. We conclude that elimination of any stretch of the elbow joint has no influence on the clinical reflex responses.

#### 3.1.3 Hand in lap with finger II just touching the biceps tendon

The brachioradial and biceps reflexes were examined in the right arm, with the hand in the lap, in an additional 17 patients (3 males, 14 females; median age =53 years, range: 19–79 years). When eliciting the biceps reflex, we took care not to put any pressure on the biceps tendon. When tapping the radial bone, the responses in the midway position changed when the forearm was moved to full supination, as follows: + to 0 in 14 patients and ++ to 0 in 3 patients. When tapping the biceps tendon, the responses observed in the midway position did not change substantially when the forearm was moved to full supination, as follows: + to +in 13 patients, ++ to ++ in 3 patients, and ++ to +in one patient.

#### 3.1.4 Effects of changing the forearm position on the brachioradial reflex in patients with stroke

**FIGURE 2:**
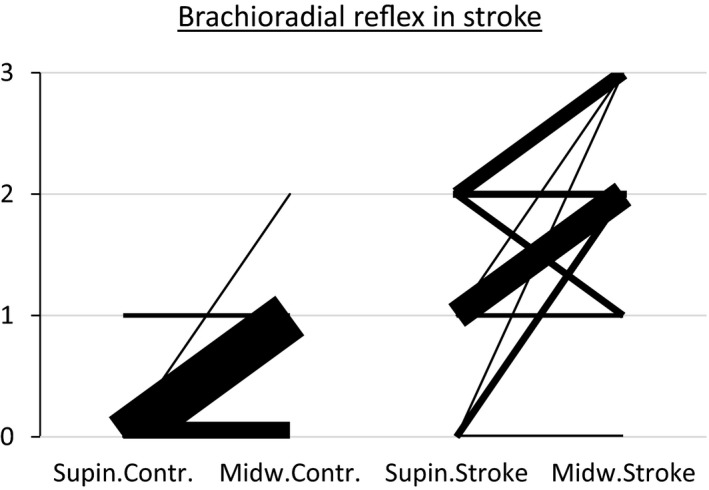
Effect on the brachioradial reflex on 32 patients with acute stroke (16) or previous stroke while the forearm was either in supination or midway position. 2: Response in control arm versus arm affected by stroke. 32 persons with either acute stroke (16) or stroke sequelae (16) to one hemisfere were tested. Brachioradial reflex was elicited while position of the forearm was either in full supination or midway position. Response was assessed clinically and depicted in the figure as described in fig 1. The response in the unaffected control arm is depicted on the left. On the right is the response in the arm affected by the stroke, only five out of these showed 0 response on supination as opposed to all, but two on the control side. Notably, one person with acute stroke showed 0 response in both positions on the “stroke” side, while the response was 1(+) in the midway position and 0 in supination on the control side, a response pattern seen on the control side in 21 persons altogether in this group (hence, the very wide bar)

We studied 16 patients that had experienced acute strokes and 16 patients that had experienced previous strokes (>three months after a stroke). Among these 32 patients, 13 were male and 19 were female, and the median age was 72 years (range 39–94 years). In 27/32 (84%) of these patients, in the brachioradial reflex, the biceps contraction persisted (hyperreflexia) when the arm was supinated (Figure 2). In contrast, persistence of the brachioradial reflex in supination occurred only in the contralateral control arm in 2/32 patients (Figure 2). The preservation of the biceps contraction when tapping the radius in supination was marginally higher in 15/16 patients with previous strokes versus 9/16 of those with acute strokes (*p* =.047, Fisher's exact test).

### 3.2 EMG investigations

**FIGURE 3:**
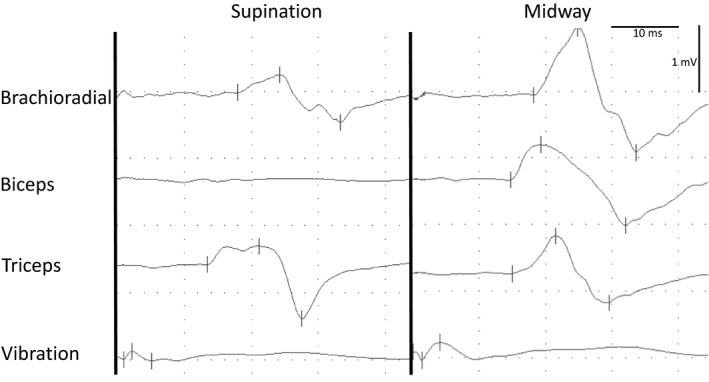
An example of the EMG examination of the brachioradial reflex from the left arm of a 48‐year‐old female control. 3: Raw surface EMG traces of the brachioradial reflex from the left arm of a 48‐year‐old female with no signs of first motor neuron disease or a peripheral neurological disorder. Calibration bars in upper right are common for all traces. Recordings were made simultaneously by surface electrodes on the three muscles (see text for detail) and a piezoelectric electrode placed on the skin over the lateral epicondyle of the humerus to record the vibration. Reflexes were elicited from the radial bone with a special reflex hammer (Dantec), attached to a cable for triggering the EMG sweep. The bone tap was performed with the forearm in full supination (left panel) and the midway position (right panel). The latency for the response was persistently longer to the brachioradial muscle compared to the biceps and triceps muscle, but only to an extend explained by the longer distance for the efferent conduction through the motor nerve fibers. EMG peak‐to‐peak amplitudes in the biceps and brahioradial muscles were persistently lower when the forearm was supinated, compared to the midway position

For the ten patients included in the EMG investigations (9 females, 1 male, median age 51, range: 38–72 years), the most common diagnoses were migraine with aura (30%) and no neurological diagnosis (30%). We observed no significant differences in the EMG latency or peak‐to‐peak amplitudes recorded on the different sides of patients. Accordingly, here, we present data for the left side, to avoid overrepresentation of the individuals. Recordings from one patient in supination and midway positions of the forearm are presented in Figure 3.

#### 3.2.1 Latencies for EMG responses

For the left brachioradial reflex, the EMG response of the biceps muscle in the midway (90°) position showed a median latency (Table 1) of 15.1 ms. This latency was marginally longer (by 1.5 ms) than the latency measured when the biceps tendon was tapped (13.6 ms). However, the difference was not significant. We observed a similar difference in the latencies for the triceps muscle EMG responses. Rotating the forearm did not influence the latencies.

**TABLE 1:** Effect of forearm position on the latencies (ms) of the left brachioradial and biceps reflexes, recorded with EMG in the biceps, brachioradial, and triceps muscles in 10 patients without a CNS lesion

Muscle	Pronation (0°)	Midway (90°)	Supination (180°)
Brachioradial reflex			
Biceps	15.5 (13.0–16.6)^b^	15.1 (12.6 – 17.6)^a^	15.6 (12.6 – 23.5^)c^
Brachioradial	18.0 (15.6–21.6)	18.2 (15.4–17.6)	18.8 (17.0–21.5)
Triceps	17.3 (15.2–20.9)	16.5 (13.9–18.9)	15.5 (13.5 to 19.0)
Biceps reflex			
Biceps	13.8 (9.7–16.3)	13.6 (11.3–15.7) ^a^	13.8 (11.6–16.8)
Brachioradial	19.0 (14.9–20.9)	18.6 (14.0–20.4)	18.5 (15.7 –20.3)^d^
Triceps	15.2 (11.7–18.4)	14.0 (12.7–18.1)	14.5 (11.1–17.1)

Notes

Values are the median (range) latencies (ms) of the responses. a: latency for brachioradial versus. biceps reflex was not significantly different; b: no EMG response was recorded in 3 subjects; c: no EMG response was recorded in one subject; d: no EMG response was recorded in one subject.

### 3.3 Quantitative measurement of the EMG response

The peak‐to‐peak amplitudes of the EMG responses (in mV) are shown in Table 2. The EMG responses to tapping the radial bone (i.e., the brachioradial reflex) were markedly reduced, when the arm position was changed from the midway position to full supination. These responses were reduced by ~80% in the biceps muscle (*p* <.001) and by ~75% in the brachioradial muscle (*p* <.01). Similar, but less pronounced effects were observed in the brachioradial muscle, when the position was changed from midway to full pronation (*p* <.01). No significant changes were observed in the triceps with changes in arm position.

**TABLE 2:** Effect of forearm position on the peak‐to‐peak (mV) amplitudes of left brachioradial and biceps reflexes, recorded with EMG in the biceps, brachioradial, and triceps muscles in 10 patients without a CNS lesion

Muscle	Pronation (0°)	Midway (90°)	Supination (180°)
Brachioradial reflex			
Biceps	0.5 (0.0–1.6)	1.1 (0.5–1.5)	0.2 (0.0–0.7)^a^
Brachioradial	1.0 (0.6–1.7)^b,c^	2.4 (0.8–5.1)	0.6 (0.4–0.9)^b,c^
Triceps	0.5 (0.4–1.2)	1.0 (0.5–2.2)	0.6 (0.2–3.1)
Biceps reflex			
Biceps	0.5 (0.2–1.3)	1.0 (0.0–1.7)	0.6 (0.3–4.3)
Brachioradialis	0.5 (0.3–0.8)	0.6 (0.1–1.4)	0.5 (0.2–0.9)
Triceps	0.5 (0.2–0.6)	0.6 (0.1–1.7)	0.4 (0.3–1.3)

Notes

Values are the median (range) peak‐to‐peak amplitudes (mV) of the responses. Statistical evaluation: a, supination versus midway, *p* < .001; b, pronation and supination versus midway, *p* < .01; c, pronation versus supination, *p* < .01.

When the biceps tendon was tapped (i.e., for the biceps reflex), the EMG responses in all three muscles were independent of forearm rotations.

Quantitative measurements of the vibration of the humerus were performed by the piezoelectric electrode in response to tapping the radius (Figure 3) or the biceps tendon.

Since the variations of the peak‐to‐peak amplitude were considerable, we included the results of both the right and left arm: For the brachioradial reflex, the calculated % mean of the vibration amplitude was 86% for supination, 116% for midway, and 98% for pronation. For the biceps, the similar values were 77%, 115%, and 108%, none of these values showed significant differences of the transmitted vibration.

## 4 DISCUSSION

### 4.1 Some methodological aspects of the study

We investigated the clinical features of the biceps and brachioradial reflexes in subjects in whom brachial reflexes were preserved, demonstrated by a tap of the radius, which resulted in a visible biceps muscle contraction. Because it is very difficult to rate brachioradial muscle contractions clinically, we used the extent of biceps contraction to rate the brachioradial reflex.

For a more objective evaluation of the reflexes, we performed an EMG investigation in 10 patients with the forearm placed on a custom‐made table. We used the same setup for these 10 patients to compare the results obtained with the forearm placed on the table to those obtained with the hand in the lap.

### 4.2 Clinical and neurophysiological investigations of how the reflexes were transmitted

When the brachioradial reflex was tested by tapping the distal radius in a patient sitting with the hand in the lap, we could not completely exclude a tiny stretch of brachioradial and biceps muscles caused by a tiny extension of the elbow. For example, in testing a tendon reflex in cats, the muscle only had to be extended by a minimum of 8 µm to elicit the stretch reflex (Denny‐Brown & Liddell, 1927). In man, a radial tap that caused the forearm to move a few mm downward has been shown to result in a 0.2 mm elongation of the biceps muscle (Hoffmaan, 1922). This small extension is likely to occur during routine testing of the brachioradial reflex, either with a hand in the lap or with the patient lying supine with the forearm resting on the abdomen.

In the present study, when the forearm and hand were placed on a custom‐made table, extension of the elbow was impossible. This prevented stretching the two muscles involved: the brachioradial and biceps muscles. In this position, the EMG response in the biceps was delayed by ~2 ms (mean difference) after a radial tap compared to a tap on the biceps tendon. This difference was marginal, but not significant, and a similar delay (a few ms) was observed in a previous study (Lance, 1965). This delay was consistent with the speed of a vibration wave, 450 m/s, in human bone in vivo (Cheng et al., 1989).

In contrast, we estimated that a response mediated through an afferent nerve from the distal radius would be delayed by at least 4–5 ms. This estimate was based on the velocity of highly myelinated fibers (50–60 m/s (Stetson et al., 1992)) and a length of the radius of 240–280 mm. Consequently, the relatively short delay we observed strongly suggested that a phasic vibration through the forearm, rather than an afferent nerve impulse, elicited the biceps contraction. Similarly, the biceps contraction elicited by a tap on the vertebral border of the scapula (Wartenberg, 1945) was most likely caused by a vibration wave conducted from the bone to the muscle.

In the present study, we confirmed that full supination of the forearm obliterated the brachioradial reflex (Wartenberg, 1945; Westbury, 1972). In this position, after the radial tap, the responses in the biceps and brachioradial muscles disappear (clinical) or decrease (EMG), but not after tapping the biceps tendon. The quantitative measurements of the transmitted vibration showed no significant difference between the positions, but since the variations in the measurements were considerable, the data are not powered to exclude a moderate difference.

A potential explanation for the disappearance of the brachioradial reflex in supination might be that, in the absence of tension in the muscle, the muscle spindles do not respond to tonic vibration (Hoffmann, 1922; Karam, 1977). In contrast, high conduction velocity afferent nerves have low thresholds in response to stretch; in fact, they typically discharge even when the muscle is slack (Lance, 1965). The different response of the muscle spindles in a relaxed muscle and in a muscle under tension led to the following prediction; when the biceps muscle is nearly totally relaxed in forearm supination, a vibration propagating through the bone will result in no reflex contraction, but the effect of a tap on the biceps tendon will not be changed with supination. Notably, this result was also observed in the subgroup that received no pressure on the tendon. Thus, the different reflex contractions observed in the biceps muscle in response to the two stimuli strongly indicate that vibration is the stimulus for the biceps contraction after a tap on the radius.

In contrast, our results suggest that the stimulus for eliciting the biceps reflex, a typical deep tendon reflex, is most likely a sudden stretch. Indeed, we found that a tap on the tendon, without any prepressure on it, resulted in a contraction of the biceps muscle, independent of the forearm position.

Similarly, in testing the brachioradial reflex, the biceps contraction also disappeared in the supinated forearm in the 10 patients tested with EMG, with the hand in the lap, and subsequently, with the forearm placed on a table. These results suggest that similar mechanisms must underlie the brachioradial reflex in these two setups; that is, the stimulus is a phasic vibration.

Most likely, both stretch and phasic vibration can stimulate the deep tendon reflex, either isolated or in combination, depending on the examination technique. This hypothesis is supported by a study in cats, which suggested that the same population of motor neurons responded to both maintained stretch and high‐frequency vibration (Wartenberg, 1945).

### 4.3 Clinical implications of the clinical examination of the brachioradial reflex

The results of the present study have a few implications for the clinical setting. First, the disappearance of the brachioradial reflex with the forearm in full supination occurred in 95% of healthy subjects. Therefore, in the clinical setting, the brachioradial reflex should be tested with the forearm in both the midway and the fully supinated positions. If the reflex does not disappear in supination, one should consider whether there might be other signs of a first motor neuron disorder.

Second, we found that the lack of the biceps muscle contraction disappearance could serve as a sign for stroke with 84% (27/32) sensitivity in the arm with hyperreflexia in patients that experienced a stroke. However, it should be noted that the reflex tended to be present during supination more frequently in patients with a previous stroke than in patients with acute strokes. This difference was probably due to increased spasticity in the former group. The finding of persistence of biceps contraction in supination after a radial tap in stroke patients should preferably be reproduced in a larger stroke patient population. Similar investigations should also be performed in other patients group with first motor neurons diseases in order to establish definitely the usefulness of this sign.

A final remark based on Lances’ observations and supported by the authors clinical experience: when a radial tap results in a visible reflex contraction in the biceps muscle (or an elbow flexion), it is not necessary also to tap the biceps tendon to test the biceps reflex arc (Lance & de Gail, 1966). Instead, it is suggested to repeat the radial tap in supination.

## 5 CONCLUSION

Our findings suggest that the argument as to whether deep tendon reflexes are elicited in man by stretch or phasic vibration is probably futile, because both of these stimuli can elicit these myotatic reflexes. The dependency of the brachioradial reflex on the position of the forearm should be taken into account, when this reflex is examined clinically.

## CONFLICT OF INTEREST

None declared.

### PEER REVIEW

The peer review history for this article is available at https://publons.com/publon/10.1002/brb3.2191.

## Data Availability

The data that support the findings of this study are available from the corresponding author upon reasonable request.
